# Pneumonia and Mortality Risk in Continuous Ambulatory Peritoneal Dialysis Patients with Diabetic Nephropathy

**DOI:** 10.1371/journal.pone.0061497

**Published:** 2013-04-09

**Authors:** Feng He, Xianfeng Wu, Xi Xia, Fenfen Peng, Fengxian Huang, Xueqing Yu

**Affiliations:** Department of Nephrology, The First Affiliated Hospital, Sun Yat-sen University, Guangzhou, China; University of Sao Paulo Medical School, Brazil

## Abstract

**Background:**

Although clinical experience suggests that patients with diabetes mellitus are more susceptible to several types of infections, the overall scope of pneumonia in continuous ambulatory peritoneal dialysis (CAPD) patients with diabetic nephropathy (DN) has received little attention.

**Methods:**

This was a prospective observational cohort study in CAPD patients in which prognostic risks of pneumonia were evaluated in DN and non-DN patients by Cox regression analysis. Hazard ratios of pneumonia events, all-cause and pneumonia-related mortality were calculated by Kaplan-Meier curves and the Cox proportional hazards model for DN versus non-DN patients.

**Results:**

A total of 1148 patients (58.6% male, 48.34±15.78 years) had a median follow-up of 23.8 months and a maximum follow-up of 72.0 months. The pneumonia incidence rate of 62.3/1,000 patient-years in CAPD patients with DN was significantly higher than that of 28.5/1,000 patient-years in non-DN patients. On multivariate analysis, independent predictors of pneumonia occurrence in CAPD patients with DN were high body mass index (hazard ratio [HR], 1.15; 95% confidence interval [CI], 1.01–1.31; P = 0.037) and low serum albumin level (HR, 0.87; 95% CI, 0.78–0.98; P = 0.014). Older age (HR, 1.63; 95% CI, 1.35–1.96; P<0.001) was an independent risk factor for the presence of pneumonia in non-DN patients. CAPD patients with DN had higher pneumonia-related mortality (HR, 4.424; 95% CI, 1.871–10.461; P<0.001) and all-cause mortality (HR, 2.608; 95% CI, 1.890–3.599; P<0.001) hazards than their non-DN counterparts, even when extensive demographics, comorbidities, and lab adjustments were made.

**Conclusions:**

The pneumonia and all-cause mortality risks were strikingly higher in CAPD patients with DN than in non-DN counterparts, which may warrant further investigation and therapeutic care intensification.

## Introduction

Registry studies typically rank infection second to cardiovascular disease as a cause of death in dialysis patients, and approximately one in every five infection-related deaths is attributed to pulmonary causes [1]. Regarding pneumonia, the mortality rate of pulmonary infection in hemodialysis (HD) patients has been reported to be 14- to 16-fold higher than in the general population [2]. In another study, the cumulative probability of pneumonia hospitalizations at 5 years was 36% in hemodialysis patients [3]. Although hemodialysis patients have an increased risk of pulmonary infection, which contributes to sizeable morbidity and mortality, the overall scope of pneumonia in continuous ambulatory peritoneal dialysis patients has received little attention.

Patients with diabetes mellitus (DM) are considered to be more susceptible to several types of infections, including pneumonia, urinary tract infection, and skin infection [4], [5]. Diabetic patients may have increased susceptibility to pneumonia due to hyperglycemia, increased risk of aspiration, decreased immunity, pulmonary microangiopathy, impaired lung function, and coexisting morbidity [6]. Moreover, diabetic nephropathy (DN) may occur in approximately 40% of patients with DM and has become the leading cause of end-stage renal disease (ESRD), which may further impair patient ability to combat pneumonia due to a chronic uremic milieu, older age, and the presence of comorbidities [3], [7], [8]. To the best of our knowledge, few if any studies have attempted to estimate the incidence, risk factors, and prognosis of pneumonia in CAPD patients with diabetic nephropathy.

Therefore, the objective of the current study was to define the clinical features and outcomes of pneumonia in CAPD patients. Specifically, we wished to define: (1) the incidence rate of pneumonia, (2) the prognostic risks of pneumonia events, and (3) the association with mortality in CAPD patients with DN.

## Materials and Methods

### Study design and participants

This was a prospective, observational cohort study of patients recruited from a single peritoneal dialysis (PD) center of the First Affiliated Hospital of Sun Yat-sen University. Enrollment occurred from February 1, 2006 to February 1, 2011 and follow-up extended to February 1, 2012, including a total of 1148 patients older than 18 years who had received CAPD for >3 months. Patients were followed every 3 months after the start of CAPD until death or censoring, which occurred because of a transfer to a non-participating dialysis center, withdrawal from the study, kidney transplantation, at the end of the follow-up period on February 1, 2012, or at a maximum follow-up of 6 years. We divided patients into DN and non-DN group according to diabetic nephropathy. For all participants, the total number of episodes of pneumonia infection and the date of the first episode were recorded. The occurrence of pneumonia was determined from Part A Medicare in-patient claims, using the following International Classification of Diseases, Ninth Revision, Clinical Modification codes: (1) viral pneumonia, 480.x. and 484.1; (2) pneumococcal pneumonia 481; (3) other bacterial pneumonia 482.xx and 483.x; (4) fungal pneumonia 112.4, 114.0, 115.xx, 484.6, 484.7; (5) pneumonia caused by other or unspecified organisms 482.89, 482.9, 483.8, 484.3, 484.5, 484.8, 485, and 486 [3]. We defined our main outcome of pneumonia as presence of one of these diagnostic codes and exhibiting signs of infection on chest x-rays. The endpoint of follow-up was pneumonia-related mortality or all-cause mortality. The study design was approved by the Clinical Research Ethics Committee of the First Affiliated Hospital of Sun Yat-sen University. All participants provided their written informed consent before inclusion.

### Data collection and laboratory measurements

Baseline demographic data and clinical data such as age, gender, diabetes, diabetic nephropathy (with proven biopsy, or an urinary albumin: creatinine ratio (ACR)>30 mg/g), hypertension, history of cardiovascular disease (myocardial infarction, ischemic stroke, or limb amputation due to peripheral arterial disease), and history of stroke were collected at the start of CAPD treatment. Diabetes was defined on the basis of diabetes mellitus registered as a primary kidney disease or as a comorbid condition [9]. Hypertension was recorded if the patient was taking antihypertensive drugs or had two separated measured blood pressures ≥140/90 mmHg. Stroke was defined as evidence of an acute disturbance of focal neurological function with symptoms lasting>24 hours and considered to be due to intracerebral hemorrhage or ischemia [10].

Baseline biochemical parameters were collected 3 months after the start of CAPD and included blood pressure, hemoglobin, serum albumin, C-reactive protein, total triglycerides, total cholesterol, HDL-C, LDL-C, plasma urea, and plasma creatinine. All parameters were measured in the biochemical laboratory of the First Affiliated Hospital of Sun Yat-sen University. Renal function was calculated using the mean creatinine and urea clearance, adjusted for body surface area (mL/min/1.73 m^2^), and renal function was calculated every 3 months. All PD patients selected peritoneal dialysis as the initial dialysis, and no patient was anuric when initiating the study. Patients were interviewed by trial nurses for general condition and concomitant medication information monthly in person or by telephone.

### Statistical analysis

Variables are presented in this study as mean ± SD, median (interquartile range), or number (proportion) where appropriate. Differences in variables between the DN and non-diabetic nephropathy (non-DN) group were tested using the Student's t-test, the non-parametric Mann-Whitney test, or the chi-square test where appropriate. Timeline incidence data were analyzed using a Poisson model. Life tables were used to calculate cumulative proportions surviving during follow-up. Cox regression analysis was used to evaluate the prognostic risks of the presence of pneumonia in the DN and non-DN group at baseline. Two different approaches were applied: (1) univariate Cox regression analysis was selected from the following variables: age, body mass index, hypertension, cardiovascular disease, stroke, diastolic blood pressure (DBP), mean artery pressure (MAP), estimated glomerular filtration rate (eGFR), C-reactive protein, and serum albumin for the presence of pneumonia, which were statistically different in baseline parameters comparison between two groups, and (2) the multivariate Cox regression analysis model using covariates by a backward stepwise selection procedure (entry: P≤0.05; removal: P>0.1, the selection criterion was from acquiesce in SPSS software system as well as the importance of clinical concern). Time-to-event analysis of pneumonia events, all-cause mortality and pneumonia-related mortality was performed using Kaplan-Meier survival curves, the Log-Rank test and the Cox proportional hazards model for the DN group compared with non-DN group. Multivariate models were constructed sequentially using only the group, then adding demographic characteristics (age at enrollment, gender, and BMI), then adding comorbidities (hypertension, cardiovascular disease, and stroke), then adding eGFR, and finally adding laboratory values (serum albumin, HDL-C, and LDL-C), in addition, other parameters exclusion by collinearity. Statistical significance was defined as P<0.05 using two-tailed tests. Statistical analyses were performed using SPSS 13.0 for Windows (SPSS, Chicago, IL, USA).

## Results

### Patient characteristics at baseline

The demographic and clinical characteristics of CAPD patients are summarized in Table 1, and categorized according to diabetic nephropathy. A total of 1148 patients were enrolled in this study (age 48.34±15.78 years, male 58.6%), with a median follow-up of 23.8 months and a maximum follow-up of 72.0 months, including 190 (16.6%) patients with DN (DN group: age 60.13±11.12 years, male 56.3%), and 958 (83.4%) patients without DN (non-DN group: age 46.02±15.53 years, male 59.1%). Reasons for exclusion were lost to follow-up (n = 25), transfer to hemodialysis (n = 60), renal transplantation (n = 151), transfer to other centers (n = 35), and poor compliance (n = 8). Compared with non-DN patients, DN patients more frequently had hypertension, cardiovascular disease, and stroke, as well as presented with a higher BMI, eGFR, and CRP, but a lower DBP, MAP, and serum albumin. However, no significant differences in baseline parameters were found between diabetic nephropathy patients with (n = 103) and without biopsy (n = 87).

**Table 1:** Baseline characteristics of 1148 CAPD patients.

Characteristics	Total	DN	Non-DN	P value
Demographics				
All, n(%)	1148(100.0)	190(16.6)	958(83.4)	
Age (years)	48.34±15.78	60.13±11.12	46.02±15.53	<0.001
Male, n(%)	673(58.6)	107(56.3)	566(59.1)	0.531
Body mass index (kg/m^2^)	21.20±3.42	22.32±3.84	20.99±3.33	<0.001
Comorbidities, n(%)				
Diabetes mellitus	262(22.8)	190(100)	72(7.5)	<0.001
Hypertension	753(65.6)	144(75.8)	609(63.6)	0.002
Cardiovascular disease	79(6.9)	38(20.0)	41(4.3)	<0.001
Stroke	89(7.8)	28(14.7)	61(6.4)	<0.001
Blood Pressure				
SBP (mmHg)	130.94±20.38	137.93±22.86	136.93±19.85	0.563
DBP (mmHg)	84.70±14.55	75.48±12.61	88.55±14.21	<0.001
MAP (mmHg)	101.14±17.84	96.30±14.48	102.11±18.28	<0.001
eGFR (mL/min per 1.73m^2^)	7.97±3.42	9.23±3.64	7.73±3.40	<0.001
Laboratory data				
Hemoglobin (g/L)	95.01±22.85	97.20±20.67	94.57±23.24	0.119
Serum albumin (g/L)	36.80±5.69	34.28±4.75	37.31±5.73	<0.001
C-reactive protein (mg/L)^a^	1.94 (0.76–7.18)	2.98 (1.00–10.47)	1.70 (0.73–6.53)	<0.001
Total triglycerides (mmol/L)	1.79 ± 1.38	1.82 ± 1.16	1.73 ± 1.42	0.335
Total cholesterol (mmol/L)	5.08±2.60	5.28±1.50	5.04±2.77	0.096
HDL-C (mmol/L)	1.26±0.58	1.22±0.52	1.27±0.59	0.317
LDL-C (mmol/L)	2.93±1.33	2.99±1.06	2.92±1.38	0.516

Data expressed with a plus/minus sign are the mean±SD.

a Median (interquartile range).

Abbreviations: CAPD, continuous ambulatory peritoneal dialysis; SBP, systolic blood pressure; DBP, diastolic blood pressure; MAP, mean artery pressure; eGFR, estimated glomerular filtration rate; HDL, high-density lipoprotein-C; LDL-C, low-density lipoprotein.

### Characteristics of pneumonia patients

**Figure 1:**
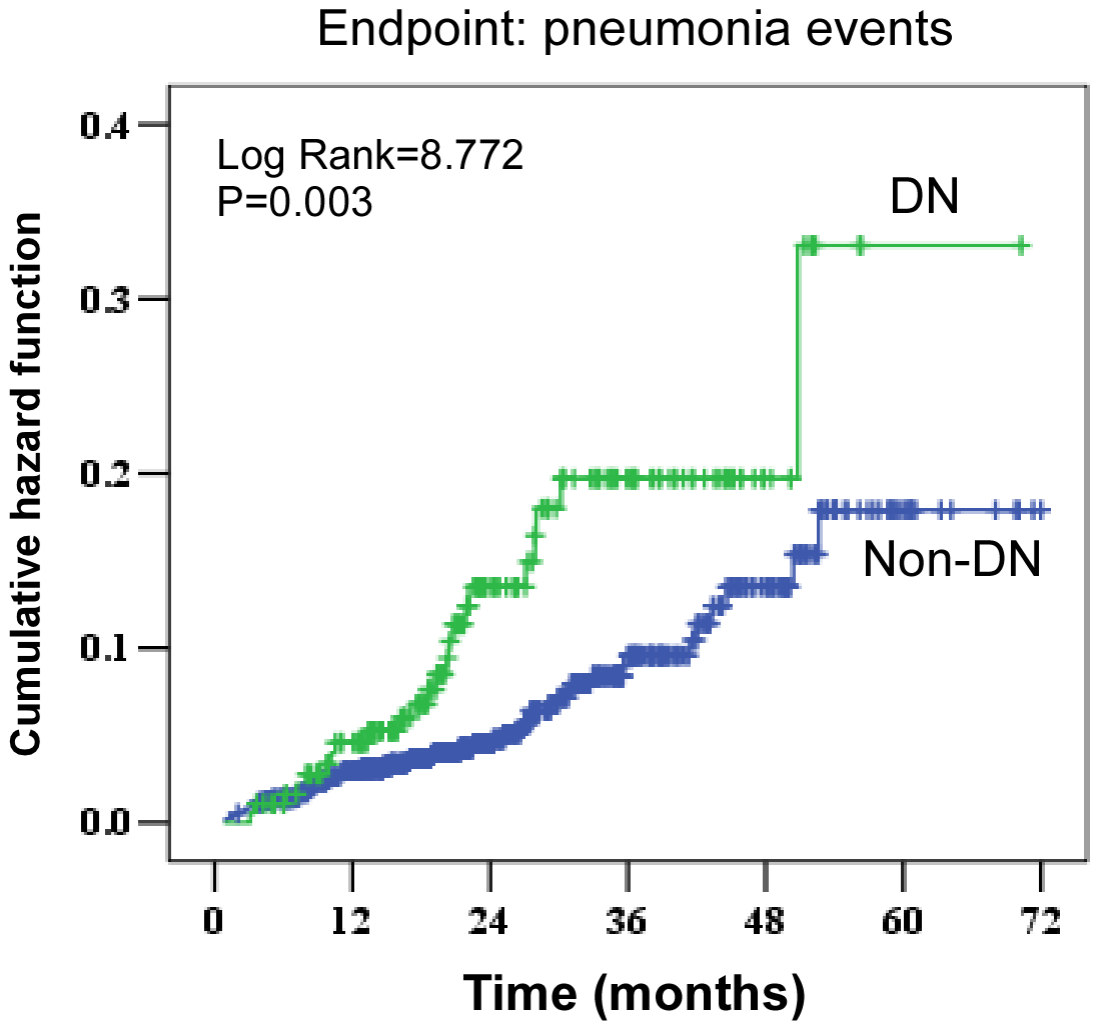
Cumulative incidence of pneumonia events according to diabetic nephropathy.

The characteristics of pneumonia patients are shown in Table 2. During the study period, a total of 78 pneumonia events occurred in all patients, including 24 events in the DN group and 54 events in the non-DN group. Pneumonia incidence rates were 12.63% higher in the DN group than that of 5.63% in the non-DN group (P = 0.001). The overall incidence rate of pneumonia was 34.3/1,000 patient-years (62.3/1,000 patient-years in the DN group and 28.5/1,000 patient-years in the non-DN group, P<0.001). Obviously, the cumulative hazard of developing pneumonia was significantly higher in the DN group than that in the non-DN group (HR, 2.176; 95% CI, 1.344–3.522; P = 0.003) (Figure 1). Additionally, The cumulative pneumonia survival was 87%, 82%, and 68% at 1, 3, and 5 years, respectively, in the DN group compared with 96%, 88%, and 83% at 1, 3, and 5 years, respectively, in the non-DN group. Furthermore, patients with DN had a higher BMI (23.60±1.80 vs. 20.99±2.99; P = 0.001), a stroke more frequently (12.5% vs. 11.1%; P = 0.049), decreased DBP (71.91±8.56 vs. 82.00±13.15; P = 0.001), decreased MAP (92.42±8.95 vs. 99.71±14.70; P = 0.009), decreased eGFR (7.29±2.96 vs. 9.69±5.13; P = 0.013), and decreased serum albumin (33.03±4.54 vs. 37.60±4.99; P<0.001) than patients without DN. However, there was no difference with regard to age or C-reactive protein level between the two groups.

**Table 2:** Characteristics of pneumonia patients with and without diabetic nephropathy.

Characteristics	DN	Non-DN	P value
Demographics			
All, n(%)	24 (30.8)	54 (69.2)	
Age (years)	60.13±11.12	46.02±15.53	0.074
Male, n(%)	16 (66.7)	25 (46.3)	0.156
Body mass index (kg/m^2^)	23.60±1.80	20.99±2.99	0.001
Comorbidities, n(%)			
Diabetes mellitus	24(100)	7(13.0)	<0.001
Hypertension	19(79.2)	40(74.1)	0.843
Cardiovascular disease	3(12.5)	1(1.9)	0.158
Stroke	3(12.5)	6(11.1)	0.049
Blood Pressure			
SBP (mmHg)	133.46±15.11	135.13±21.99	0.743
DBP (mmHg)	71.91±8.56	82.00±13.15	0.001
MAP (mmHg)	92.42±8.95	99.71±14.70	0.009
eGFR (mL/min per 1.73m^2^)	7.29±2.96	9.69±5.13	0.013
Laboratory data			
Hemoglobin (g/L)	94.51±14.43	99.57±24.37	0.257
Serum albumin (g/L)	33.03±4.54	37.60±4.99	<0.001
C-reactive protein (mg/L)^a^	1.76(0.69–11.42)	3.02(1.28–8.40)	0.432
Total triglycerides (mmol/L)	1.90±1.38	1.84±1.12	0.216
Total cholesterol (mmol/L)	4.73±1.04	5.25±1.12	0.079
HDL-C (mmol/L)	1.29±0.35	1.33±0.55	0.116
LDL-C (mmol/L)	2.70±0.69	2.99±1.01	0.158

Data expressed with a plus/minus sign are the mean±SD.

a Median (interquartile range).

Abbreviations: SBP, systolic blood pressure; DBP, diastolic blood pressure; MAP, mean artery pressure; eGFR, estimated glomerular filtration rate; HDL-C, high-density lipoprotein; LDL-C, low-density lipoprotein.

### Independent predictors of pneumonia

Clinical and laboratory variables that were statistically different in Table 1 were included in the Cox regression analysis. Table 3 shows the predictors of pneumonia for CAPD patients with DN. There were no significant variables using univariate Cox regression analysis, however, the multivariate analysis model identified higher BMI and lower serum albumin as independent predictors of pneumonia occurrence after adjusting for age, hypertension, cardiovascular disease, stroke, DBP, MAP, and eGFR. The predictors of pneumonia for the non-DN group are summarized in Table 4. Significant variables included age and DBP by univariate Cox regression analysis. Only older age remained significant in the multivariate model after adjusting for BMI, hypertension, cardiovascular disease, stroke, DBP, MAP, eGFR, CRP, and serum albumin, which showed that every 10-year increase in age increased the risk of a pneumonia event by 63% (HR, 1.63; 95% CI, 1.35–1.96; P<0.001).

**Table 3:** Predictor variables and multivariate model for pneumonia events in patients with diabetic nephropathy.

Predictors	Hazard ratio	95% CI	P value
Predictor variables			
Age (per 10-year age increase)	1.27	0.87–1.87	0.215
Body mass index (per 1 kg/m^2^ increase)	1.08	0.96–1.22	0.185
Hypertension (yes/no)	1.45	0.54–3.90	0.459
Cardiovascular disease (yes/no)	0.60	0.18–2.01	0.407
Stroke (yes/no)	0.77	0.23–2.60	0.674
DBP (per 1 mmHg increase)	0.98	0.95–1.01	0.133
MAP (per 1 mmHg increase)	0.98	0.91–1.01	0.182
eGFR (per 1mL/min per 1.73m^2^ increase)	1.00	0.90–1.13	0.883
C-reactive protein(per 1 mg/L increase)	0.98	0.91–1.05	0.539
Serum albumin (per 1g/L increase)	0.93	0.86–1.01	0.066
Multivariate model^a^			
Body mass index (per 1 kg/m^2^ increase)	1.15	1.01–1.31	0.037
Serum albumin (per 1g/L increase)	0.87	0.78–0.98	0.014

Abbreviations: DBP, diastolic blood pressure; MAP, mean artery pressure; eGFR, estimated glomerular filtration rate; CI, confidence interval.

a Adjusted for variables from the above predictor variables using a backward stepwise cox proportional hazards model with a stay criterion of 0.10. P<0.05 represents statistical significant.

**Table 4:** Predictor variables and multivariate model for pneumonia events in patients without diabetic nephropathy.

Predictors	Hazard ratio	95% CI	P value
Predictor variables			
Age (per 10-year age increase)	1.51	1.27–1.80	<0.001
Body mass index (per 1 kg/m^2^ increase)	1.00	0.92–1.09	0.938
Hypertension (yes/no)	1.72	0.94–3.17	0.081
Cardiovascular disease (yes/no)	0.35	0.05–2.54	0.300
Stroke (yes/no)	1.75	0.75–4.10	0.195
DBP (per 1 mmHg increase)	0.97	0.95–0.99	0.003
MAP (per 1 mmHg increase)	0.99	0.98–1.00	0.071
eGFR (per 1mL/min per 1.73m^2^ increase)	0.93	0.85–1.03	0.146
C-reactive protein(per 1 mg/L increase)	1.00	0.98–1.03	0.852
Serum albumin (per 1g/L increase)	1.00	0.95–1.05	0.937
Multivariate model^a^			
Age (per 10-year age increase)	1.63	1.35–1.96	<0.001

Abbreviations: DBP, diastolic blood pressure; MAP, mean artery pressure; eGFR, estimated glomerular filtration rate; CI, confidence interval.

a Adjusted for variables from the above predictor variables using a backward stepwise cox proportional hazards model with a stay criterion of 0.10. P<0.05 represents statistical significant.

### Pneumonia and survival

Fatal events were registered during follow-up. A total of 164 patients died and 21 deaths were pneumonia related. In terms of all-cause death, 57 occurred in the DN group and 107 in the non-DN group. In terms of pneumonia-related deaths, 10 occurred in the DN group and 11 in the non-DN group. Furthermore, the cumulative pneumonia-related mortality was 4%, 10%, and 23% at 1, 3, and 5 years, respectively, in the DN group compared with 1%, 4%, and 6% at 1, 3, and 5 years, respectively, in the non-DN group. Meanwhile, the cumulative overall mortality at 1, 3, and 5 years was 25%, 50%, and 58%, respectively, in the DN group compared with 9%, 24%, and 33%, respectively, in the non-DN group.

### Mortality analyses

**Figure 2:**
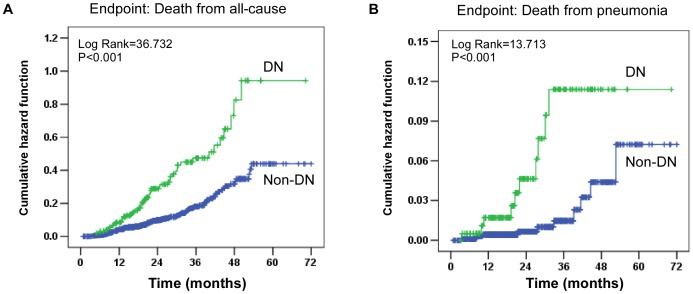
Cumulative incidences of all-cause and pneumonia-related death according to diabetic nephropathy. (A) Cumulative hazard of all-cause death. The HR in the DN, as compared with non-DN, was 2.608 (95% CI, 1.890–3.599). (B) Cumulative hazard of pneumonia death, for which the HR in the DN group was 4.424 (95% CI, 1.871–10.461).

**Figure 3:**
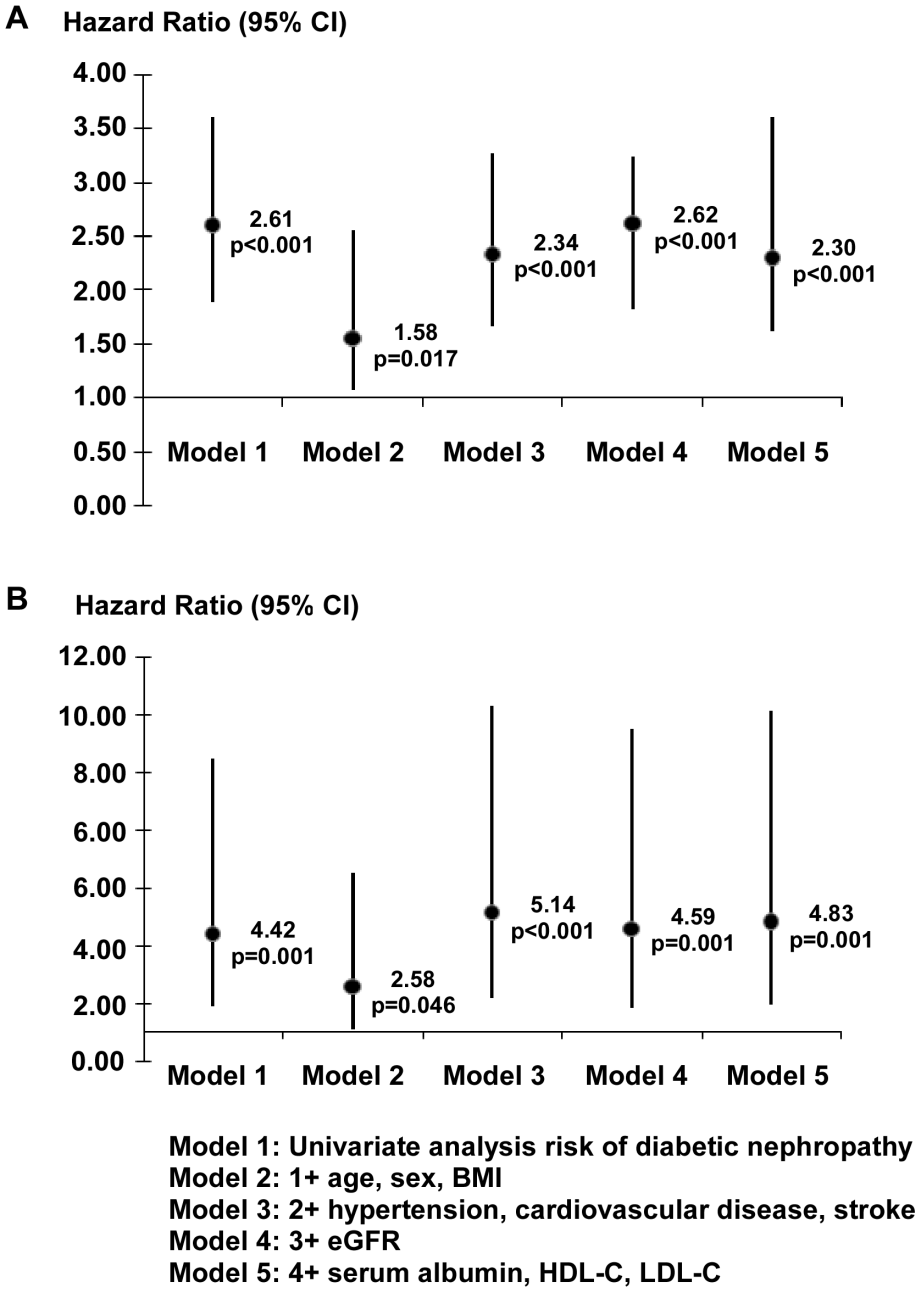
The hazard ratios of diabetic nephropathy on CAPD for the prediction of all-cause and pneumonia-related mortality. (A) All-cause mortality and (B) pneumonia-related mortality by diabetic nephropathy. BMI, body mass index; eGFR, estimated glomerular filtration rate; MAP, mean artery pressure; CRP, C-reactive protein; HDL-C, high-density lipoprotein; LDL-C, low-density lipoprotein; HR, hazard ratio; CI, confidence interval.

Compared with non-DN patients, DN patients were at increased risk of all-cause and pneumonia-related mortality based on Kaplan-Meier curves and Cox regression analysis (Figure 2). In univariate analysis risk of diabetic nephropathy, the cumulative hazard was significantly higher in the DN group. Specifically, it was 2.608 (95% CI, 1.890–3.599; P<0.001) for all-cause mortality and 4.424 (95% CI, 1.871–10.461; P<0.001) for pneumonia-related mortality. Furthermore, the association between DN and all-cause or pneumonia-related mortality was studied by multivariate Cox analysis (Figure 3). Similar to the univariate analysis of model 1, this elevated risk of all-cause mortality persisted after adjustment for various potential confounders, whereas adjustments for age, gender, and BMI in model 2 slightly decreased the strength (HR,1.581; 95% CI, 1.082–2.226; P = 0.017). There was still a statistically significant association found between DN and the risk of pneumonia-related mortality by additional adjustment for various confounders. In a full model including demographics, comorbidities, and labs, the adjusted HR was 4.831 (95% CI, 1.927–12.109; P = 0.001).

## Discussion

This prospective study of a single peritoneal dialysis center offers a detailed evaluation of the epidemiology, clinical features, and outcomes of pneumonia in CAPD patients. The main findings are that (i) CAPD patients with DN had a higher incidence of developing pneumonia than non-DN patients; (ii) risk factors for pneumonia occurrence were higher BMI and lower serum albumin in DN patients during CAPD, whereas older age was an independent predictor of the incidence of pneumonia in non-DN patients; and (iii) all-cause and pneumonia-related mortality rates were significantly higher in CAPD patients with DN.

The clinical epidemiology of pneumonia in dialysis patients has received comparatively little attention to date. The United States Renal Data System shows that in dialysis patients pulmonary infections account for about 115 hospital admissions per 1000 patient-years of risk [1]. Other observational studies suggest that nosocomial infections, including pneumonia, are much more common in hospitalized dialysis patients than in their non-dialyzed counterparts [11]. Guo et al. reported a pneumonia rate of 29.0 episodes per 100 patient-years in hemodialysis patients [12], which was higher than the rate of hospitalization for pneumonia as the primary diagnosis (18.2 per 100 patient-years) in peritoneal dialysis patients. For comparison with our population, we collected all pneumonia incidences (diagnosed by chest x-rays exhibiting signs of infection in the lungs), including hospital and community-acquired pneumonia. We found the overall pneumonia rates were 34.3/1,000 patient-years, and pneumonia rates in the DN patients were 62.3/1,000 patient-years, as well as 28.5/1,000 patient-years in the non-DN patients. There may be several explanations for the lower prevalence of pneumonia in our population than in the data reported previously in dialysis patients. On the one hand, we applied a uniform and relatively strict standard in diagnosing pneumonia by chest x-rays, which may have lead to us missing some cases. On the other hand, our participants were mainly from Guangdong province located in southern China where the climate is so mild that the occurrence of pneumonia is not very prevalent. Interestingly, we found that the CAPD patients with DN were more susceptible to develop pneumonia. Importantly, we found the hazard of developing pneumonia for CAPD patients with DN was significantly higher than the patients without DN.

Diabetes is frequently associated with multiple complications, such as hypertension, ischemic heart disease, left ventricular hypertrophy, arrhythmia, diabetic nephropathy, arteriosclerosis obliterans, diabetic retinopathy, hyperglycemia, and dyslipidemia, which exist even before the pre-dialysis stage [13]. In the present study, CAPD patients with DN were older, and more commonly had comorbidities of hypertension, a cardiovascular condition (chronic heart or cerebrovascular disease), and stroke. Moreover, we found that pneumonia patients with DN were significantly associated with higher BMI and lower serum albumin, which also were independent predictors of pneumonia occurrence even when extensive demographics, comorbidities, and lab adjustments were made. Our results agree with the report which showed that low levels of serum albumin associated with greater pneumonia risk in dialysis patients [12]. Obviously, obesity (measured as a BMI) increases the risk for diabetes and is also an independent risk factor for ESRD [14]–[16]. Likewise, our findings are partially consistent with large studies (of >1000 participants) among incident dialysis patients with a long-term follow-up (5–10 years) which showed that high BMI was associated with worse dialysis outcomes and increased mortality risk among peritoneal dialysis and hemodialysis patients [17]–[19]. Therefore, intensive management of risk factors (high BMI and low albumin) and prompt recognition of infection is therefore recommended for CAPD patients with DN to improve quality of life. In addition, an independent risk factor in our study for the presence of pneumonia in non-DN patients was older age. A similar observation was previously made among older patients [20], [21].

Sarnak and Jaber reported that pulmonary infection mortality rates are 14–16 times higher in dialysis patients than in the general population [2]. In our study, the pulmonary mortality of 5.3% found in CAPD patients with DN, was five times higher than in non-DN patients. Meanwhile, the cumulative hazard was 4.831 (95% CI, 1.927–12.109; P = 0.001) for pneumonia-related mortality when adjustments were made for extensive demographics, comorbidities, and labs in DN patients after an episode of pneumonia. A similar trend was observed for all-cause mortality. According to the report, patients with diabetes mellitus are considered to be immunocompromised and therefore more susceptible to infections [22]. ESRD is widely understood to be a state of inflammatory, endothelial, and redox dysfunction [23]–[25]. Micro-inflammation is a pivotal contributor to the pathogenesis of cardiovascular disease [26]. Therefore, DN patients on CAPD may be chronically immunosuppressed, by virtue of the uremic internal milieu and the very frequent coexistence of serious comorbid medical conditions. Pneumonia typically leads to ‘macro’-inflammation. Standard indices of inflammatory activity were not available in this study. Thus, our hypothesis that pneumonia and subsequent mortality reflect sudden increases in inflammatory activity remains speculative.

This study was limited in that it was a prospective observational study and pneumonia was identified based on results of a chest x-ray test, which might miss some pneumonia patients. A substantial number of pneumonia cases likely occurred after admission to the hospital with another serious illness. In this scenario, an association between pneumonia and mortality could reflect the occurrence of this other serious illness, without a direct causal link between pneumonia and death. Our study involved a prevalent cohort of CAPD patients; therefore, patients were not studied at a uniform phase of their chronic kidney disease. Smoking and microbiologic test data were not used because of incomplete information.

Despite its limitations, our study may have practical implications. We found that higher BMI and lower serum albumin are independent predictors for pneumonia in CAPD patients with DN, which suggests that these patients should pay attention to weight control but avoid malnutrition. Furthermore, both the burden of disease and mortality associations were noteworthy. These findings might encourage the use of preventive measures, timely diagnosis of pneumonia, and prompt initiation of appropriate treatment. Prospective mechanistic, observational, and therapeutic research is greatly needed in this population.
